# Pasting, thermo, and Mixolab thermomechanical properties of potato starch–wheat gluten composite systems

**DOI:** 10.1002/fsn3.1506

**Published:** 2020-03-25

**Authors:** Fen Xu, Wei Liu, Qiannan Liu, Chunjiang Zhang, Honghai Hu, Hong Zhang

**Affiliations:** ^1^ Institute of Food Science and Technology Chinese Academy of Agricultural Sciences/Comprehensive Key Laboratory of Agro‐products Processing Ministry of Agriculture Beijing China; ^2^ Hefei CAAS Nutridoer Co. Ltd. Academy of Food Nutrition and Health Innovation Chinese Academy of Agricultural Sciences Hefei China

**Keywords:** dough properties, gelatinization, microstructure, potato starch, rheology, wheat gluten

## Abstract

This research investigated the viscosity, thermal, thermomechanical, and microstructural properties of potato starch–wheat gluten composite systems with different starch/gluten ratios under mechanical shear and heating conditions. Results showed that the peak, trough, and final viscosities increased with the increase in potato starch fraction. The breakdown and setback values of samples decreased with increasing gluten content, and the endothermic enthalpy showed a similar variation trend. The gelatinization temperature of the samples increased significantly as the gluten proportion increased. Morphological observation showed that the gluten protein was wrapped around potato starch granules and the starch granules have a diluting effect on gluten network. Moreover, gluten formed a water diffusion barrier out of the starch granules, this barrier effect and the competitive hydration between starch and gluten could primarily explain the delayed gelatinization temperatures.

## Graphical abstract

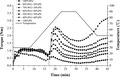

## 1 INTRODUCTION

Potato (*Solanum tuberosum* L.) is the world's fourth most important food crop after rice, wheat, and maize. Potato is also one of the main raw materials for starch extraction, and the starch content of potato is generally 9%~25%. Both the whole potato flour and the extracted potato starch are widely used in food industry due to their unique physicochemical properties. Studies showed that potato flour (potato starch as main composition) or potato starch supplements significantly influence the dough's processing performance (i.e., rheological, thermal, and thermomechanical properties) and the final products quality (i.e., color, texture, nutrition, and flavor) (Zhang, Xu, Wu, Hu, & Dai, 2017). For instance, adding 20% potato flour to wheat flour will maintain the rheological properties and improve the nutritional value of the noodle and steamed bread (Liu, Mu, Sun, Zhang, & Chen, 2016; Xu, Hu, Dai, Hu, Dai, & liu, Q., Huang, Y., & Zhang, H., 2017; Xu, Hu, Liu, Dai, & Zhang, 2017). Potato starch at a level of 5%–15% is often used to improve the textural properties of wheat‐based alkaline instant noodles (Noda et al., 2006; Zaidul, Yamauchi, Matsuura‐Endo, Takigawa, & Noda, 2008). Potato starch has high water‐binding capacity and swelling power, which differ from those of other commercial starches such as corn, wheat, and rice starches (Zhang, Lim, & Chung, 2019). Compared with starches from other botanical sources, potato starch contains larger quantities of phosphate groups which can hydrate quickly under hydrothermal treatment, thus has a higher viscosity and forms a more clearly gel (Noda et al., 2006; Yusuph, Tester, Ansell, & Snape, 2003). These gelling and pasting properties of potato starch can be affected by environmental conditions; such physicochemical properties were considered to significantly influence dough formation and the final products quality (Chung et al., 2014; Gryszkin, Zięba, Kapelko, & Buczek, 2014; Hong, Chen, Zeng, & Han, 2016; Huang, Zhou, Jin, Xu, & Chen, 2016).

Although the effect of potato flour and potato starch on dough formation has been widely studied, most of the tested dough systems contain a variety of ingredients (i.e., wheat starch, potato starch, potato protein, and wheat gluten), making it difficult to know the effects of starch on gluten network formation. A common simplifying approach in investigations of the role of gluten and starch in dough systems is to use gluten–starch model dough samples (recombine the extracted starch and gluten). This allows precise control of the gluten content and also excludes complex effect of other ingredients in the dough systems (Jekle, Mühlberger, & Becker, 2016; Zhang, Mu, & Sun, 2018). Particularly, the interaction between wheat starch and wheat gluten was investigated by using starch–gluten model dough system. Research showed that gluten can form 3D matrices through protein–protein and protein–carbohydrate interactions when gluten is subjected to certain external conditions of mixing and heating; such interactions also largely affect the functional qualities of flour products (Bock & Damodaran, 2013; Singh & Singh, 2013). Starch granules in dough systems act as filler particles within gluten matrix. Previous works have also explored the interaction between starch and protein from other different perspectives (Espinosa‐Dzib, Ramírez‐Gilly, & Tecante, 2012; Homer, Kelly, & Day, 2014; Ravindra, Genovese, Foegeding, & Rao, 2004). Dutta reported that silk sericin and rice starch can be thermo‐mechanically conjugated and casted into continuous 2D films through evident molecular interaction between starch and sericin (Dutta, Dutta, & Devi, 2018). Both starch, protein, and their interaction are responsible for the macroscopic properties of food matrix (Jekle et al., 2016).

However, as the two main ingredients, the function of potato starch and wheat gluten in potato‐based staple foods still need to be studied. Understanding the physicochemical properties of potato starch–wheat gluten composite systems and their interactions under thermal and mechanical treatments would help researchers to achieve better designs of the formulation and processing conditions of food systems, in which these two biopolymers are the major ingredients.

## 2 MATERIALS AND METHODS

### 2.1 Raw materials

Shepody potatoes were provided by the Dingbian Science and Technology Bureau of Shaanxi Province, China. Potato starch was isolated from Shepody potatoes containing 4.24% moisture, 95.76% starch, and 1.95% ash. Wheat gluten was purchased from Tian Long Wheat Flour Co., Ltd. (Henan, China). The wheat gluten contained 7.22% moisture, 89.34% protein, 9.82% starch, and 0.82% ash. The production way of the wheat gluten was as follows: Wheat flour was mixed with water and then separated by pumping the flour paste into a series of hydrocyclone. The obtained flow was then washed and sieved to get the wet gluten. After that, a drying process was applied before packing. Moisture, protein, and ash contents were determined using AOAC official methods (Association of Analytical Chemists, 2000), and starch content was determined using a total starch assay kit (Megazyme, K‐TSTA 04/2009). All these measurements were performed in triplicate.

### 2.2 Potato starch isolation

Potato starch was obtained using the method previously reported by Gani (Gani et al., 2014). Potatoes were washed, peeled, and then ground into paste by using a blender (KM005, Kenwood, England). The resultant slurry was sieved through a muslin cloth to remove potato residues. The resultant starch suspension was left to stand overnight, and the solid residue was washed ten times with distilled water. The starch dispersion was sieved through a 150 μm mesh sieve, and the supernatant liquid was discarded. Finally, purified starch was freeze‐dried for 36 hr in a pilot‐scale vacuum freeze dryer (Genesis™ SQ, Virtis, America).

### 2.3 Experimental design

The potato starch–wheat gluten composite systems were prepared based on five different starch–gluten ratios (2:8, 3:7, 4:6, 5:5, and 6:4). Potato starch and wheat gluten were used as control samples. The thermal, microstructural, and thermomechanical properties of control samples and the potato starch–wheat gluten composite systems were studied.

### 2.4 Paste viscosity

The viscosity properties of native potato starch, wheat gluten, and starch–gluten composite systems were analyzed using Rapid Viscosity Analyser (RVA, super 4, Newport Scientific, Australia). The tests were conducted under fixed shear conditions with a completely controlled temperature cycle of heating, holding, and cooling. This procedure ensures the generation of highly reproducible gelatinization and pasting profiles. Samples (2 g each) were suspended in 25 ml of water to prepare suspensions to be measured by the RVA. The constant rotating speed (160 rpm) and fixed heating up and cooling down process were adopted for the measurement. The heating temperature was maintained at 50°C for 1 min, followed by a ramped‐up temperature to 95°C at a rate of 12°C/min; the temperature was held at 95°C for another 2.5 min and then cooled down to 50°C at the same rate (12°C/min). Finally, the temperature was held again at 50°C for 2 min. The peak viscosity, trough viscosity, final viscosity, peak time, break down, setback, and pasting temperature were recorded during the heating and subsequent cooling process.

### 2.5 Differential scanning calorimetry (DSC)

The thermal properties of potato starch, wheat gluten, and starch–gluten composite systems were investigated using a differential scanning calorimeter (TA Q200, TA Instruments, New Castle, USA). Samples (3 mg) were weighed into stainless steel pans, and 10 μl of distilled water was added before the pans were hermetically sealed. The pans were equilibrated at 4°C for 12 hr before heating from 25 to 100°C at a rate of 5°C/min by DSC. The onset temperature (T_0_), peak temperature (Tp), peak width at half height (ΔT), and endothermic enthalpy (ΔH) were calculated automatically. A sealed empty pan was used as reference.

### 2.6 Thermomechanical measurements

Mixing and pasting properties of potato starch, wheat gluten, and their mixtures were studied using a Mixolab analyzer (Chopin Technologies, Villeneuve‐la‐Garenne, France). Samples were placed in a bowl with two kneading arms, and the amount of water added was determined by the water absorption capacity of the samples. The torque (Nm) was obtained as a function of time, thus allowing evaluation of the thermochemical properties of dough samples. The Chopin^+^ standard protocol was used to determine the water absorption of flour by the dosage of water until the dough was able to reach the maximum torque of 1.1 ± 0.05 Nm (equivalent to 500 Farinograph units). The quality of protein network and the starch behavior of dough were analyzed using the standard procedure during heating and cooling. First, the mixture was held at 30°C for 8 min. Second, the temperature was increased to 90°C at a rate of 4°C/min and then maintained for another 8 min. Finally, the temperature was decreased to 50°C at a rate of 4°C/min and held for 10 min. Parameters including water absorption (%), maximum torque (C1), minimum torque (C2), peak viscosity (C3), holding viscosity (C4), final viscosity (C5), protein weakening (C1–C2), breakdown (C3–C4), setback (C5–C4), and gelatinization temperature were obtained from the recorded curve.

### 2.7 Environmental scanning electron microscopy (ESEM)

The microstructures of the raw potato starch, wheat gluten, and starch–gluten composite dough samples were observed through field‐emission ESEM (Quanta 200 FEG, FEI, USA). The dough samples used for ESEM observation were obtained using the Mixolab mixing bowl. The blended flour and water were added in the bowl and mixed at a constant temperature of 30°C, and the mixing rate was 80 rpm/min. The amount of added water was determined by Mixolab until the torque produced by the dough reached 1.1 ± 0.05 Nm at 14% moisture basis. The dough samples used for microstructural observation were obtained from the inner part of the dough. The samples were then placed in 3% glutaraldehyde overnight, washed with 0.1 mol/L phosphate buffer thrice, and dehydrated in a graded acetone series (25%, 50%, 75%, 80%, and 100%) for 20 min. Afterward, the samples were dried and sprayed with gold. Potato starch granules and wheat gluten were used for observation without pretreatment. ESEM was used to observe and photograph the samples at 500× magnification.

### 2.8 Statistical analysis

The data reported were averages of triplicate observations and expressed as mean ± standard deviation. ANOVA and Tukey's test at .05 significance level were conducted to evaluate the significant differences among sample means. ANOVA was performed using SPSS 18.0 (SPSS Inc., Chicago, USA).

## 3 RESULTS AND DISCUSSION

### 3.1 Viscosity properties

**Figure 1:**
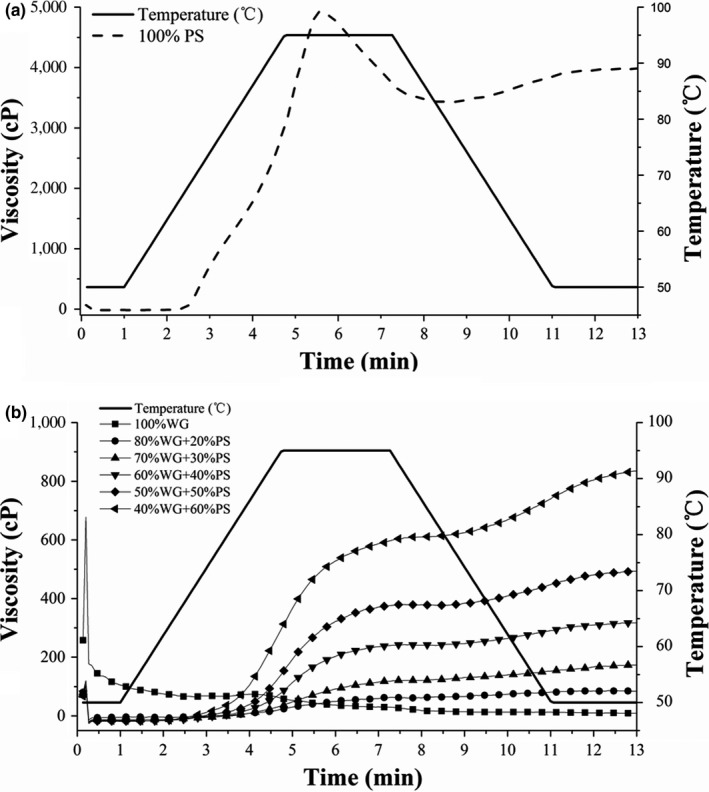
The RVA curves for potato starch (PS, a), wheat gluten (WG), and PS–WG composites (b) with different starch fractions

The pasting profiles for potato starch, wheat gluten, and their composite systems at different starch/gluten ratios were examined during heating and cooling periods by RVA. As shown in Figure 1, the pasting curves with higher content of potato starch were obviously higher than that of the samples with lower content of potato starch. The peak viscosity of potato starch–wheat gluten composite pastes decreased as the protein fraction increased. Table 1 shows that with the increasing amount of wheat gluten, the peak viscosity, trough, and final viscosities of the starch–gluten pastes decreased significantly. The decrease in the viscosity profile is likely attributed to the dilution of starch component in the starch–gluten composite pastes by gluten substitution. The result indicates that potato starch significantly contributes to the viscosity of the potato starch–wheat gluten mixed pastes, whereas the existence of gluten decreases the sample viscosity. Similar results were observed in previous studies (Baxter, Blanchard, & Zhao, 2004; Kim, Kee, Lee, & Yoo, 2014; Marcoa & Rosell, 2008). Moreover, the final viscosity was mainly contributed by the aggregation of the amylose molecules; this decrease indicated a decreasing tendency of starch paste stability and gel hardness (Sun, Gong, Li, & Xiong, 2014).

**Table 1:** RVA parameters of potato starch (PS), wheat gluten (WG), and PS–WG composites with different starch fractions

Samples	Peak (cP)	Trough (cP)	Breakdown (cP)	Final viscosity (cP)	Setback (cP)	Peak time (min)	Pasting temperature (°C)
100%WG	209.50 ± 150.61^c^	9.50 ± 0.71^d^	200.00 ± 151.32^b^	12.50 ± 4.95^f^	3.00 ± 5.66^e^	1.07 ± 0.00^c^	N.D
80%WG + 20%PS	52.50 ± 9.19^c^	45.00 ± 7.07^d^	7.50 ± 2.12^c^	76.50 ± 10.61^f^	31.50 ± 3.54^de^	6.97 ± 0.05^a^	N.D
70%WG + 30%PS	120.50 ± 7.78^c^	99.00 ± 8.49^d^	21.50 ± 0.71^bc^	176.50 ± 4.95^e^	77.50 ± 3.54^cde^	7.00 ± 0.00^a^	N.D
60%WG + 40%PS	227.00 ± 12.73^c^	198.50 ± 12.02^cd^	28.50 ± 0.71^bc^	304.50 ± 17.68^d^	106.00 ± 5.66^cd^	7.00 ± 0.00^a^	N.D
50%WG + 50%PS	373.00 ± 2.83^b^	331.50 ± 9.19^c^	41.50 ± 6.36^bc^	494.00 ± 1.41^c^	162.50 ± 7.78^c^	7.00 ± 0.00^a^	N.D
40%WG + 60%PS	592.00 ± 7.07^b^	538.50 ± 7.78^b^	53.50 ± 0.71^bc^	839.50 ± 6.36^b^	301.00 ± 1.41^b^	7.00 ± 0.00^a^	87.65 ± 0.07^a^
100%PS	5,168.00 ± 335.17^a^	3,578.50 ± 208.60^a^	1589.50 ± 126.57^a^	4,057.50 ± 102.53^a^	479.00 ± 106.07^a^	5.60 ± 0.00^b^	66.75 ± 0.07^b^

Note

Different superscript letters in each column indicate significant differences (*p* < .05).

*N*.D, Not detected.

Breakdown is the reflection of the susceptibility of cooked starches to their disintegration during heating (Yang et al., 2016). With increasing wheat gluten content, the breakdown viscosity decreased significantly. This effect would considerably aid the formation of potato starch–wheat gluten composite pastes. Setback is mainly derived from the recrystallization of amylase, but it can also be affected by the presence of proteins (Bucsella, Takacs, Vizer, Schwendener, & Tomoskozi, 2016). In the present study, the setback decreased significantly with increasing wheat gluten content. Table 1 displays that the peak time of the formation of the potato starch–wheat gluten composite pastes was around 7 min, which is significantly longer than the control samples. The pasting temperature of the potato starch paste was 66.75°C, whereas that of the starch–gluten composite pastes with a 60% starch fraction was 87.65°C. This result indicates that the addition of gluten increased the pasting temperature of the starch–gluten composite pastes. Similar findings were observed in the lentil starch–lentil protein and wheat starch–wheat gluten mixtures (Jekle et al., 2016; Joshi, Aldred, Panozzo, Kasapis, & Adhikari, 2014). This phenomenon might be ascribed to the hydrophilic property of gluten; its strong water absorption capacity decreases the content of available water for potato starch to swell and gelatinize. Therefore, the pasting temperature increased. The RVA instrument was employed most frequently to measure the viscosity of starch, and the absence of pasting temperatures of samples with starch fractions below 60% in this study was mainly attributed to the limitations of the instrument.

### 3.2 Thermal properties

**Figure 2:**
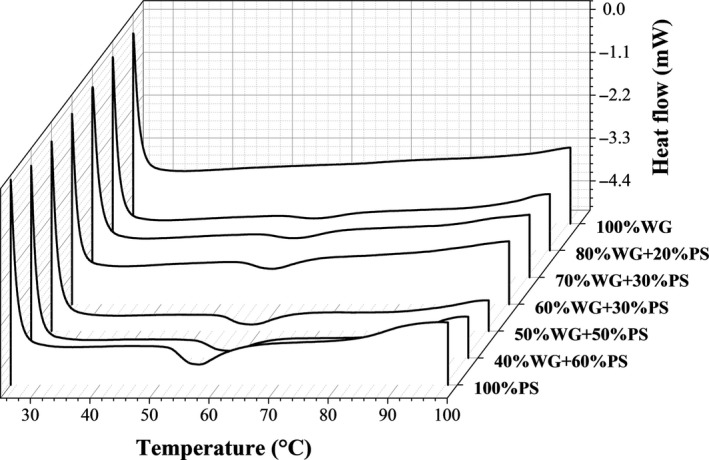
The DSC thermograms of potato starch (PS), wheat gluten (WG), and PS–WG composites with different starch fractions

The DSC thermograms of potato starch, wheat gluten, and their composites with different starch fractions were shown in Figure 2. The phase transition peaks of all the samples ranged between 50 and 70°C, and such peaks were believed to be associated with starch gelatinization. No peak was observed of the wheat gluten sample within the tested temperature range. A thermodynamic transition occurred in the starch–gluten composite systems during the heating process. When water was added, the potato starch began to swell. Under heat treatment, the starch molecules began to acutely vibrate, and the intermolecular hydrogen bond was broken followed by the disappearance of the starch crystalline region. In this process, the state of the starch molecules changed with the alteration of energy. With the increase of wheat gluten fraction in potato starch–wheat gluten composites, obvious decrease in the areas under the thermal transition peak above the extrapolation can be observed and the DSC curves tend to be flat with the addition of wheat gluten.

The gelatinization parameters of samples are listed in Table 2. The gelatinization onset (T_0_) and peak temperatures (T_p_) of the native potato starch were 51.64°C and 56.51°C, respectively, and the endothermic enthalpy was 12.10 J/g. With the increasing proportion of gluten, the onset and peak temperatures increased significantly, whereas the endothermic enthalpy (ΔH) decreased. This finding may be attributed to the reassociation of starch to a certain extent and the gluten addition significantly changed the thermal denaturation process of the complex. The absorption of excess water by gluten interfered with starch swelling and thus delayed the gelatinization process (Champenois, Rao, & Walker, 1998; Jekle et al., 2016; Lennart & Ann‐Charlotte, 1986; Yang, Song, & Zheng, 2011). Moreover, the gluten exerts a structural effect on gelatinization intensity because gluten can disturb the contact area between disintegrated starch granules during heating (Jekle et al., 2016).

**Table 2:** DSC parameters of potato starch (PS), wheat gluten (WG), and PS–WG composites with different starch fractions

Samples	T_0_ (°C)	T_P_ (°C)	△H (J/g)	△T (°C)
100% WG	—	—	—	—
80%WG + 20%PS	54.82 ± 1.21^ab^	59.20 ± 0.34^b^	2.50 ± 0.36^cd^	7.28 ± 0.49^a^
70%WG + 30%PS	54.40 ± 0.01^ab^	59.11 ± 0.04^b^	2.63 ± 0.26^cd^	6.22 ± 0.49^a^
60%WG + 40%PS	54.06 ± 0.04^b^	58.88 ± 0.02^b^	3.92 ± 0.01^bc^	6.43 ± 0.17^a^
50%WG + 50%PS	53.63 ± 0.00^b^	58.43 ± 0.06^bc^	4.58 ± 0.08^bc^	6.23 ± 0.05^a^
40%WG + 60%PS	53.41 ± 0.14^b^	58.07 ± 0.11^c^	6.01 ± 0.62^b^	6.05 ± 0.18^a^
100% PS	51.64 ± 0.74^c^	56.51 ± 0.06^d^	12.10 ± 2.67^a^	6.88 ± 0.04^a^

Note

Different superscript letters in each column indicate significant differences (*p* < .05).

### 3.3 Thermomechanical properties

**Figure 3:**
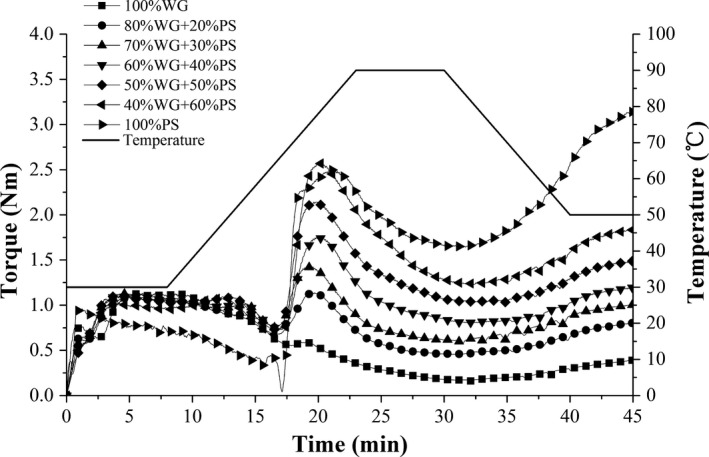
Thermomechanical curves obtained by Mixolab for potato starch (PS), wheat gluten (WG), and PS–WG composites with different starch fractions

The thermomechanical properties of samples during mixing, temperature rising, pasting, and subsequent cooling were shown in Figure 3. The rheological behavior of samples during dough development was measured by a small‐scale mixer with two mixing blades and recorded constantly to generate torque versus time curves. Such measurement process is equivalent to the determination of dough characteristics in the whole process of converting powder into products (Huang et al., 2010). Samples with different potato starch/wheat gluten ratios showed significant differences in their thermomechanical curves. Parameters obtained from the curves were shown in Table 3. Results showed that the water absorption capacity of the potato starch–wheat gluten composite dough samples increased obviously with increasing gluten content. This result confirmed that the gluten protein demonstrates a stronger water absorption capacity than starch. The peak torque value (C1) of potato starch sample was 0.16 Nm and failed to reach 1.1 ± 0.05 Nm. This is mainly due to the limitations of the instrument on the analysis of starch‐rich substances. The peak (C3), holding (C4), and final viscosities (C5) increased with the increasing potato starch content in the dough samples. This result indicates that potato starch plays a dominant role in dough viscosity. Moreover, the stability and the retrogradation properties of the composite dough samples increased with the increasing addition of potato starch. Breakdown (C3–C4) and setback (C5–C4) values also increased with the increasing content of potato starch. These results are consistent with the previously described findings measured by RVA. The lack of obvious difference in the minimum torque (C2) observed between samples indicated that the weakening property of the protein did not change significantly. This effect was mainly achieved because the changes in torque caused by gluten were masked by the starch changes with the increase in temperature. The gelatinization temperature of wheat gluten sample was 58.8°C, whereas that of the potato starch sample was 51.6°C. The samples containing larger amounts of gluten displayed higher gelatinization temperatures. This changing trend was also consistent with the previous result obtained by RVA. Gluten's water absorption capacity was stronger than that of starch; hence, the competitive hydration between polymers hindered the swelling and gelatinization processes of potato starch. However, the gelatinization temperatures of the samples obtained by Mixolab were not exactly the same with the temperature obtained by RVA. This discrepancy might be ascribed to the different magnitudes of the imposed deformation between the two methods (Kim et al., 2014).

**Table 3:** Mixolab parameters on potato starch (PS), wheat gluten (WG), and PS–WG composites with different starch fractions

Mixolab parameters	100% WG	20% PS	30% PS	40% PS	50% PS	60% PS	100% PS
Water absorption (%)	109.10	93.20	86.30	80.10	74.20	70.00	65.00
Maximum torque (C1, Nm)	1.14	1.10	1.13	1.11	1.11	1.09	0.16
Minimum torque (C2, Nm)	0.55	0.74	0.75	0.73	0.72	0.68	0
Peak viscosity (C3, Nm)	0.60	1.14	1.43	1.76	2.14	2.57	4.12
Holding viscosity (C4, Nm)	0.17	0.45	0.57	0.81	1.03	1.23	2.72
Final viscosity (C5, Nm)	0.39	0.80	0.99	1.19	1.48	1.82	5.22
Protein weakening (C1–C2, Nm)	0.59	0.36	0.38	0.38	0.39	0.41	0.16
Breakdown (C3–C4, Nm)	0.43	0.69	0.86	0.95	1.11	1.34	1.40
Setback (C5–C4, Nm)	0.22	0.35	0.42	0.38	0.45	0.59	2.50
Gelatinization temperature (°C)	58.80	54.60	55.10	53.40	52.40	51.60	51.60

### 3.4 Morphological analysis

**Figure 4:**
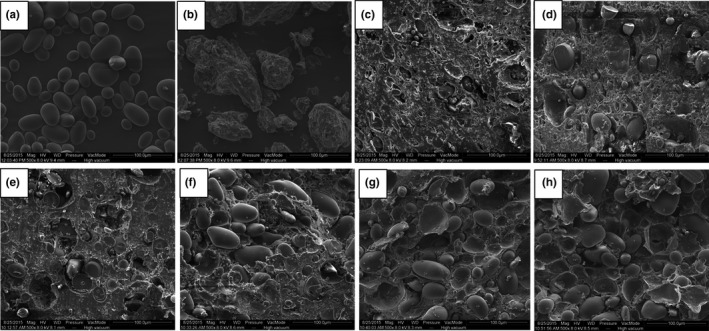
Scanning electron micrographs of native potato starch granules (a), wheat gluten (b), and PS–WG composite dough samples with different starch fractions (c: 0%, d: 20%, e: 30%, f: 40%, g: 50%, and h: 60%)

The pristine surfaces of the native potato starch granules and wheat gluten were observed through ESEM (Figure 4a and b). The images clearly show that the wheat gluten condensed into blocks, and almost no starch granule can be observed. The potato starch granules were characterized by an oval or elliptical shape with a smooth surface. Similar morphological features also observed in previous studies (Lovedeep, Narpinder, & Navdeep, 2002). The average particle size of potato starch in this study is 26.4 μm (calculated by Image J software). This result is very close to a previous report of 25.8 μm (Alvani, Qi, Tester, & Snape, 2011). The size difference may caused by the potato variety differences (Jaspreet & Narpinder, 2001).

Figure 4 (c–h) shows the microstructures of wheat gluten and potato starch–wheat gluten composite dough samples. As is shown in Figure 3c, the gluten formed a continuous network structure and exhibited a distinguishable gluten film structure through the interaction of molecules. Some small hollows or ditches can be observed in the gluten dough. When different proportions of potato starch granules were mixed with gluten, starch granules were embedded in the gluten network and formed new structures. As displayed in the images (Figure 4d–h), the incorporation of starch in gluten caused the structures of the composite dough samples to disaggregate with thin fibril‐like gluten compared with the compact structure of the control sample. The addition of the potato starch decreased the continuity of the gluten coating on the dough surfaces, and the starch granules were not completely wrapped and appeared less embedded in the gluten matrix with higher content of potato starch. Previous observations on the ultrastructure of starch–gluten model dough systems showed that gluten represents continuous phase around the starch granules, forming a barrier for the starch flowability. Compared with wheat starch–gluten dough, the potato starch–gluten dough showed less consistent and uniform dough matrix (Zhang, Mu, & Sun, 2019). In addition, the gluten was wrapped around the starch granules, thus forming a barrier for the hydration of the starch granules. The diffusion of water into the starch granules was hindered by the barrier effect; this may explain the delayed gelatinization temperature of starch–gluten composites as previously measured by RVA and DSC. The competitive hydration between wheat gluten and potato starch also plays an important role in the delayed gelatinization temperature because gluten demonstrates a stronger water absorption capacity than starch as previously measured by Mixolab. When additional potato starch granules were exposed, the barrier effect of gluten was decreased and gelatinization temperature was lower.

## 4 CONCLUSIONS

The ratio of potato starch to wheat gluten has significant effect on viscosity, thermal, and Mixolab thermomechanical properties of the starch–gluten composites. Potato starch plays a dominant role in upholding paste viscosity, and gluten considerably contributes to the formation of potato starch–wheat gluten composite dough system. The ΔH value of the composites decreased significantly with the increasing gluten/starch ratio. The gelatinization temperature of the starch–gluten composite systems increased with the addition of wheat gluten. Wheat gluten enveloped the surface of starch granules and potato starch granules showed a diluting effect on gluten network. The current findings are expected to contribute to the better understanding of the mixing, gelling, and pasting behavior of potato starch–wheat gluten composite systems. Such information is particularly relevant to the development of potato‐based staple foods. For example, the viscosity of potato‐based staple products may increase in the heating process with the increase of potato proportion. Less cooking time may be needed for the potato‐based products with higher potato proportion since higher starch content lead to a decrease of gelatinization temperature. Moreover, a proper proportion of potato starch/flour was suggested in potato‐based staple products since potato starch granules have a diluting effect on gluten network.

## CONFLICT OF INTEREST

The authors declare that they do not have any conflict of interest.

## ETHICAL APPROVAL

This study does not involve any human or animal testing.
